# In utero and peripubertal metals exposure in relation to reproductive hormones and sexual maturation and progression among boys in Mexico City

**DOI:** 10.1186/s12940-020-00672-0

**Published:** 2020-11-25

**Authors:** Pahriya Ashrap, John D. Meeker, Brisa N. Sánchez, Niladri Basu, Marcela Tamayo-Ortiz, Maritsa Solano-González, Adriana Mercado-García, Martha M. Téllez-Rojo, Karen E. Peterson, Deborah J. Watkins

**Affiliations:** 1 Department of Environmental Health Sciences, University of Michigan School of Public Health, 1415 Washington Heights, Ann Arbor, MI 48109 USA; 2 Department of Biostatistics, University of Michigan School of Public Health, Ann Arbor, MI USA; 3 Faculty of Agricultural and Environmental Sciences, McGill University, Montreal, Quebec, Canada; 4 Center for Nutrition and Health Research, Instituto Nacional de Salud Pública, Cuernavaca, Morelos Mexico; 5 Mexican Council for Science and Technology, Mexico City, Mexico; 6 Department of Nutritional Sciences, University of Michigan School of Public Health, Ann Arbor, MI USA

**Keywords:** Metal, Hormone, In utero exposure, Pregnancy, Puberty

## Abstract

**Background:**

Endocrine disrupting chemicals (EDCs) such as metals have been reported to alter circulating reproductive hormone concentrations and pubertal development in animals. However, the relationship has rarely been investigated among humans, with the exception of heavy metals, such as Pb and Cd. Our aim was to investigate measures of in utero and peripubertal metal exposure in relation to reproductive hormone concentrations and sexual maturation and progression among boys from the Early Life Exposure in Mexico to Environmental Toxicants (ELEMENT) cohorts.

**Methods:**

Our analysis included 118 pregnant women and their male children from the ELEMENT study. Essential and non-essential metals were measured in urine collected from the mothers during the third trimester of pregnancy and their male children at 8–14 years. Reproductive hormone concentrations [serum testosterone, estradiol, dehydroepiandrosterone sulfate (DHEA-S), inhibin B, and sex hormone-binding globulin (SHBG)] were measured in blood samples from the children at 8–14 years. We also assessed Tanner stages for sexual maturation (genital, pubic hair development, and testicular volume), at two time points (8–14, 10–18 years). We used linear regression to independently examine urinary metal concentrations in relation to each peripubertal reproductive hormones adjusting for child age and BMI. Generalized estimation equations (GEEs) were used to evaluate the association of in utero and peripubertal metal exposures with sexual maturation and progression during follow-up based on Tanner staging and testicular volume.

**Results:**

In utero and prepubertal concentrations of some urinary metals were associated with increased concentrations of peripubertal reproductive hormones, especially non-essential metal(loid)s As and Cd (in utero), and Ba (peripubertal) as well as essential metal Mo (in utero) in association with testosterone. More advanced pubic hair developmental stage and higher testicular volume at the early teen visit was observed for boys with higher non-essential metal concentrations, including in utero Al and peripubertal Ba, and essential metal Zn concentration (peripubertal). These metals were also associated with slower pubertal progression between the two visits.

**Conclusion:**

These findings suggest that male reproductive development may be associated with both essential and non-essential metal exposure during in utero and peripubertal windows.

**Supplementary Information:**

The online version contains supplementary material available at 10.1186/s12940-020-00672-0.

## Introduction

In recent decades, a trend toward earlier onset of puberty among boys and girls has been described [1–9]. These trends in the timing of puberty, a period of physical and psychological development, have raised concerns regarding the potential impact of environmental factors, including endocrine disrupting chemicals (EDCs) [10–13]. Exposure to EDCs prenatally and at the prepubertal stage are thought to play a role in altered pubertal timing, possibly via their estrogenic or anti-androgenic effects and disruption of normal homeostatic control of the hypothalamic-pituitary-gonadal (HPG) or hypothalamic-pituitary-adrenal (HPA) axis [13–26].

Some metals and metalloids, such as cadmium (Cd), lead (Pb), mercury (Hg), and arsenic (As), are non-essential xenobiotics that are known to be harmful to human health [27–30]. These non-essential metal(loid)s are persistent in the environment and children’s exposure to them is nearly ubiquitous [31]. Several other metals, such as chromium (Cr), copper (Cu), manganese (Mn), molybdenum (Mo), selenium (Se) and zinc (Zn), are essential for optimal health but may be harmful at insufficient or excessive levels [32–36]. A number of metal(loid)s are reproductive toxicants and have endocrine disrupting properties which interfere with many aspects of endocrine functions through interacting with hormone secretion, transport and binding receptors as well as genomic expression and epigenetic modification [19, 37–43]. Some of these proposed mechanisms of actions are common to different metals, such as binding with estrogen receptor (Cd, As, Pb) and increasing lipid peroxidation (Pb, Hg), while others are specific, for instance, stimulation or inhibition of nuclear transcription activity (As) and inhibition of LH secretion (Pb) [44].

Many human and animal studies have focused on elucidating the reproductive effects associated with heavy metal and metalloids, such as Cd, Pb, Hg, and As. Though many studies on Cd were either cross-sectional and/or conducted on adults, there has been some consistency of findings with regard to the positive relationship between male and female Cd exposure and testosterone concentrations [45–53]. In a group of men with no occupational exposure, positive associations between blood Pb concentrations and testosterone and/or estradiol levels were reported [46, 48, 50]. In longitudinal studies of children’s reproductive development, childhood Pb exposure was related to later pubertal onset [54, 55] and delayed sexual maturation [56] in Russian boys in Chapaevsk, Russia. In contrast, we have shown early life exposure to Pb was associated with delays in pubertal development in girls but not boys in Mexico City [21, 22]. Hg was found to be associated with increased estradiol levels in both males and females from a small residential population in Cambodia [57], which was in agreement with a previous study among women with repeated miscarriages [58]. A recent study of As exposure through well water consumption in Taiwan suggested that As may impart an increased risk of erectile dysfunction through a reduction of circulating testosterone [59].

Although less attention has been given to the other metals in the past, a growing body of evidence suggests that certain essential or trace metals, including Cu, fluoride (F), Mn, Mo, and Se can also have adverse effects on male reproduction [60–63]. In addition, most reports on the male reproductive effects of metals are from experimental animal, epidemiological, and occupational studies usually involving high doses not commonly encountered by children. Due to the widespread exposure of humans and known adverse effects related to essential and non-essential metal exposure, concern is growing that low-level exposure may also adversely affect reproductive developmental outcomes in boys. Moreover, only a few studies have investigated the cross-sectional relationships in boys, with the exception of two recent longitudinal studies in Russia and Mexico [56, 63], and none have examined exposure during in utero development and subsequent hormone levels during puberty, a time at which steroid hormones play an essential role in reproductive development [11, 64–66].

Therefore, the present study assessed whether in utero and prepubertal exposure to metals at relatively low doses altered reproductive hormone levels or timing and progression of sexual maturation in boys. We extended the limited metals studied on this topic and examined both essential and non-essential metals in relation to reproductive hormone concentrations and progression of sexual maturation in boys from ages 8–14 years to 10–18 years.

## Method

### Study population

**Fig. 1:**
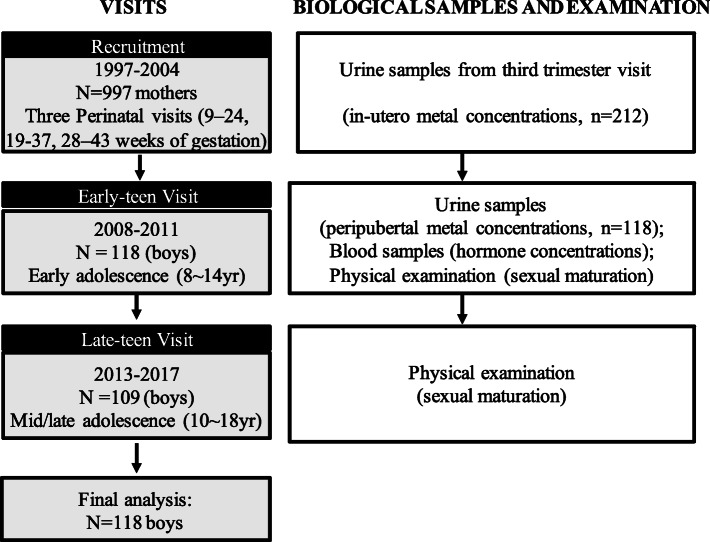
Recruitment, sample collection and examination timeline for ELEMENT cohort

Participants in this study are part of the “Early Life Exposure in Mexico to Environmental Toxicants (ELEMENT)” project, a longitudinal cohort study of pregnant women in Mexico City and their children [67]. ELEMENT recruited 997 pregnant women from maternity hospitals during their first trimester between 1997 and 2004 and their children, as previously described [21, 67–70]. Inclusion criteria included not planning to leave the area within 5 years; no history of infertility, diabetes, or psychosis; not consuming alcoholic beverages daily during pregnancy; no addiction to illegal drugs; no diagnosis of a high-risk pregnancy; being pregnant with singleton. Mothers completed interview-based questionnaires at up to three prenatal visits [mean gestational age at visit 1: 13.5 (range:9–24) weeks, visit 2: 25.1 (range: 19–37) weeks, visit 3: 34.4 (range: 28–43) weeks] and provided spot urine samples at the third trimester visit. Between 2008 and 2011, a subset of their children (*n* = 250), who were then 8–14 years of age, were contacted to participate in a follow-up study (i.e. early-teen visit). The criteria for eligibility included the availability of archived maternal biological specimens for toxicant assay interest [67]. Between 2013 and 2017, 223 (89%) of those 250 children participated in a second follow-up study at age 10–18 years (i.e. late-teen visit). Among those 223 children, 109 were boys (92% retention rate from early to late-teen visit). Figure 1 shows the study design and the timing of biological sample collection and physical examination. Participants provided spot urine and fasting blood samples, anthropometry, and reported socio-demographic information via an interviewer-administered questionnaire. In the current analyses, we included male children who finished the early-teen visit (a majority of these same boys also completed late-teen visit) and had maternal urinary metal concentration measurements and/or their early-teen visit urinary metal measurements available (*n* = 118).

### Metal concentrations

Urinary metal concentrations of 14 metals and metalloids: aluminum (Al), As, barium (Ba), Cd, cobalt (Co), Cu, iron (Fe), Mn, Mo, nickel (Ni), Pb, antimony (Sb), selenium (Se), and Zn were measured in maternal third trimester urine and urine samples collected during the early-teen visit at age 8–14 years. Prenatal and peripubertal (early-teen) urine samples were collected in sterile cups, aliquoted within 1 hour after collection, frozen and stored at − 80 °F, and shipped on dry ice to McGill University (Montreal, Canada) for analysis. Urinary metals were measured using inductively coupled plasma mass spectrometry (ICPMS; Varian 820-MS, Inc., Palo Alto, California) as described previously [71, 72]. Accuracy and precision were measured using certified reference standards (Institut National de Santé Publique du Québec, or INSPQ) with coefficients of variation (CVs) ranging from 3 to 14%, and each batch run contained procedural blanks and replicate runs [72, 73]. More details regarding quality control (QC) were previously described [73]. Values below the limit of detection (LOD) were replaced with the LOD/√2. Urinary specific gravity (SG) was measured using a handheld digital refractometer.

Pb exposure in this study was also measured in maternal patella/blood and early peripubertal blood, and results of these biomarkers in relation to pubertal development within this population have already been published [21, 22]. As patella and blood Pb concentrations are better biomarkers of long term Pb exposure, we excluded urinary Pb from the current analyses.

### Hormones

Children provided fasting blood samples during the early-teen visit at age 8–14 years. Serum aliquots were separated and frozen at− 80 °C, and then sent to the Clinical Ligand Assay Service Satellite (CLASS) Laboratory at the University of Michigan (Ann Arbor, MI) for hormone analysis. Estradiol, testosterone, inhibin B, and sex hormone-binding globulin (SHBG) were measured in serum samples as biomarkers of puberty, and dehydroepiandrosterone sulfate (DHEA-S) was measured as a biomarker of secretion of adrenal androgens. Estradiol, total testosterone, SHBG, and DHEA-S were measured using an automated chemiluminescent immunoassay (Bayer Diagnostics ACS:180). Active inhibin B was assayed using Gen II ELISA (Beckman Coulter, Webster, TX). All laboratories that performed hormone analyses employed standard quality control (QC) measures, including the use of blanks and duplicate samples to measure instrument precision and identify potential sources of contamination at different collection and measurement stages. The samples were calibrated with standards to determine the degree of bias and implement actions to prevent calibration drift. The laboratories also followed pre-specified protocols for samples that exceed QC activity control limits. Values below the LOD were replaced with the LOD/√2.

### Sexual maturation

Two pediatricians evaluated male offspring for Tanner staging and testicular volume using standardized protocols during both follow-up visits at ages 8–14 and 10–18 years. To ensure consistency, pediatricians were trained prior to the start of each follow-up as previously described [74] to evaluate Tanner staging in male participants using standardized protocols. Genital development stage (GD) was assessed as an indicator of puberty and pubic hair stage (PH) as an indicator of adrenarche, with stage 1 corresponding to no development and stage 5 corresponding to full development [75]. Right and left testicular volume (TV) were measured with an orchidometer, and the larger of the two measurements was used in analyses. As a testicular volume of 1–3 mL is considered prepubertal [76, 77] and TV ≥ 20 mL was used as an indicator of sexual maturity [77, 78], cutoffs of 3 mL and 20 mL were used to create a 3-level ordinal variable for testicular volume.

### Covariates

Covariates in our analysis included: age at the early-teen visit, BMI z-scores at both visits, and household socioeconomic status at the late-teen visit. BMI z-scores were calculated based on the World Health Organization child reference curves for age and sex (WHO, 2007) for each follow up visit. A 7-level categorical variable for socioeconomic status (SES) was estimated using a validated scale consisting of thirteen questions on housing quality, services, material goods and head of household education (Asociación Mexicana de Agencias de Investigación de Mercados y Opinión Pública, AMAI version 13 × 6) [79, 80].

### Statistical methods

The geometric mean concentrations of each metal in urine samples from pregnant women and their children were calculated. The percent of urine samples with concentrations below the LOD were reported for each metal, and metals that were detected in less than 50% of samples were excluded from further analysis.

Multiple linear regressions were used to assess associations between urinary metal concentrations and peripubertal serum hormone concentrations, where serum hormones were natural log-transformed prior to analysis to achieve normal distribution. Metal concentrations from prenatal and peripubertal urine samples were entered into regression models separately and each model was adjusted for child age and BMI z-score, and for SG as a measure of urinary dilution. Results were calculated as the percent difference in hormone (95% confidence interval) per interquartile range (IQR) increase in urinary metal concentrations. Because reproductive hormones may differ greatly in boys at pre-pubertal stages (Tanner stage = 1) vs pubertal stages (Tanner stage> 1), we performed a secondary analysis in which we examined the associations between in utero and peripubertal exposures and reproductive hormone concentrations among subjects who were pre-pubertal (pubic hair Tanner stage = 1, *n* = 94).

Longitudinal analyses were conducted to explore the association between metals and early-teen and late-teen sexual maturation parameters using repeated measures generalized estimating equation (GEE). This allows us to take fuller advantage of the data that have been collected in our longitudinal cohort study and use the additional within-person information to achieve increases in statistical power to detect associations [81]. With separate models for each metal, the GEE approach was used to fit a multinomial (ordinal) regression model for pubertal stages at each visit as a function of metal exposure, age at early-teen visit and change in time, with adjustment for potential confounders:

1 $$ g\left(E\left[{Y}_{ij}\right]\right)={\beta}_0+{\beta}_1{M}_{ij}+{\beta}_2{Age}_i+{\beta}_3{Time}_{ij}+{\beta}_4{M}_{ij}\ast {Time}_{ij}+{\beta}_5{Age}_i\ast {Time}_{ij} $$

2 $$ g\left(E\left[{Y}_{ij}\right]\right)={\beta}_0+{\beta}_1{M}_{ij}+{\beta}_2{Age}_i+{\beta}_3{Time}_{ij}+{\beta}_4{M}_{ij}\ast {Time}_{ij}+{\beta}_5{Age}_i\ast {Time}_{ij}+{\beta}_6 BMIbase+{\beta}_6 BMIvar $$

Model (1) is the crude model where *Y* is an outcome of interest (Tanner stages/testicular volume), *g* is a link function (cumulative logit), *i* denotes subject number (1, …,n), and *j* denotes visit number (1, 2). *M* is ln-transformed metal exposure, *Age* is the age at the early-teen visit*, Time* is the change in time between the early-teen and late-teen visit. Age at early-teen visit and change in time between two visits were included in the model to account for the effect of baseline age on attained Tanner stage or testicular volume, the natural pubertal progression across time, and the effect of baseline age on the natural pubertal progression. Model (2) is the final adjusted model, in which BMI z-score at the early-teen visit (*BMIbase*) and the change in BMI z-score from early-teen to late-teen visit (*BMIvar*) were included. The coefficients of interest are the cross-sectional effect of metal on tanner stage/testicular volume (β_*1*_) and the effect of metal on the progression of tanner stage/testicular volume over time (β_*4*_*).* Given the possibility that age and BMI z-score at the early-teen visit may be associated with Tanner stage and influence the future progression of Tanner stage, working independence was chosen as the covariance structure to ensure the validity of parameter estimates [82]. We ran the GEE models both with and without BMI z-score (model 1 and 2) as BMI may be on the causal pathway between exposure and puberty. However, the magnitude of estimates from both models were almost identical, therefore we reported results from the models including BMI z-score. Results are presented as odds ratios (OR) and 95% confidence intervals (95% CI) per IQR increase in exposure. As few participants were categorized as having a PH Tanner stage =3 and stage = 4 during the early-teen visit, the number of covariates we could reliably enter into models was limited [83, 84]. Thus, to minimize the number of covariates in GEE models, we included SG-corrected metal concentrations (rather than entering SG as a separate covariate which yielded similar results), using the following equation: P_c_ = P[(SG_p_ – 1)/(SG_i_ – 1)] where P_c_ is the SG corrected metal concentration (μg/L), P is the measured metal concentration, SG_p_ is the median urinary specific gravity, and SG_i_ is the individual’s urinary specific gravity.

Because SES could be a potential confounder, we ran a sensitivity analysis including SES as a covariate in models of hormones and maturation stages. Furthermore, as both low and high levels of essential metals are of concern, to explore potential non-linear associations we used adjusted generalized additive models (GAM) to graphically depict the cross-sectional relationship between metal concentrations and hormones and sexual maturation measurements. We also considered significance after adjusting for multiple testing using the Benjamini-Hochberg method [85]. Since Tanner stages/testicular volumes were not independent of each other, we calculated *q* values (adjusted *p* values) treating each outcome as a family of tests (11 tests per outcome). A cutoff of 0.15 for *q* value was used to further interpret main results with greater confidence. All analyses were performed using R version 3.5.2 and SAS 9.4.

## Results

### Demographics and exposure distributions

Characteristics of participants in the original cohort of 997 women were previously described in detail [67]. Mothers included in our analysis had similar demographic characteristics to the overall ELEMENT population; the mean age of mothers at the time of enrollment was 26.6 (standard deviation = 5.3). Mothers had on average 11 years of education, most were married or cohabitating (89%), and all lived within Mexico City. Very few (3%) reported smoking during pregnancy. Average age of the boys at the early-teen and late-teen visits were 10.4 and 13.7, respectively. Mean and standard deviation of the follow up period were 3.5 and 0.5 years. The percent of samples with metal concentrations below the limit of detection, as well as the geometric means, standard deviations, and selected percentiles of sample concentrations from prenatal and peripubertal (early-teen) visits are shown in Table 1. Spearman correlations between the prenatal and peripubertal visit metal concentrations adjusted for SG are also presented in the table. With the exception of Sb and Fe, other metals were detected in > 50% of urine samples, therefore, Sb and Fe were excluded from further analysis. Weak correlations between maternal and peripubertal metal concentrations and weak to moderate correlations between different urinary metal concentrations within maternal and child samples have been previously reported elsewhere [73].

**Table 1:** Distribution of urinary metal concentrations (μg/L) among ELEMENT mothers and their male children at age 8–14 years^a^

	In utero								Peripubertal							
	LOD	%< LOD	GM	GSD	25%	50%	75%	MAX	%<LOD	GM	GSD	25%	50%	75%	MAX	*P* value ^b^
*Essential metals*																
Co	0.4	0.0	1.2	1.9	0.8	1.2	2.1	5.2	0.0	0.7	1.5	0.6	0.8	0.9	1.6	0.63
Cu	48.2	46.3	86.1	4.4	34.1	50.2	71.9	2742	56.8	43.3	1.3	34.1	34.1	56.1	106	0.83
Mn	0.4	7.4	0.8	1.7	0.6	0.7	1.0	8.7	2.5	1.2	1.8	0.8	1.2	1.7	4.4	0.68
Mo	2.9	15.8	19.5	3.3	12.7	25.7	42.9	308	0.0	46.6	1.8	33.7	50.2	67.1	210	0.99
Se	10.5	3.2	29.9	1.7	23.0	32.6	43.1	120	1.7	47.2	1.7	36.3	53.0	65.6	141	0.26
Zn	0.1	1.2	271	2.2	160	298	451	1253	0.0	366	1.8	257	411	521	1200	0.08
*Non-essential Metals*																
Al	8.6	11.6	24.0	2.5	12.3	20.3	42.2	304	24.6	14.5	2.1	8.8	14.0	23.3	428	0.37
As	0.3	0.0	14.0	2.0	9.3	13.2	20.6	153	0.0	14.3	2.0	10.2	14.4	20.5	515	0.07
Ba	1.1	3.2	4.0	2.0	2.6	4.2	5.9	27.6	10.2	2.5	2.0	1.6	2.4	3.9	20.2	0.81
Ni	3.0	0.0	8.7	1.9	5.8	7.4	11.0	107	0.8	8.1	1.6	5.9	8.0	10.8	53.2	0.83
Cd	54.0	2.1	0.2	2.1	0.1	0.2	0.3	2.7	2.5	0.1	1.6	0.1	0.1	0.2	0.3	0.24

^a^ uncorrected for specific gravity

^b^ *P* value from Spearman correlation test between in utero and peripubertal metal concentration measurements

### Hormone and Tanner stages distributions

Distributions of reproductive hormones among ELEMENT boys were described previously [86]. Except for 12 total testosterone measurements, all measures were above the LOD. Spearman correlations among hormones were weak to moderate (R = -0.42 to 0.63). Distributions of Tanner stages of sexual maturation and testicular volume among the male children at the two follow up visits are reported (Supplementary Table S1). We additionally provided spaghetti plots depicting Tanner stage and testicular volume progression between two visits (Supplementary Fig. S1), and the distribution of measures of sexual maturation for different age groups of ELEMENT boys (Supplementary Table S2). At the early -teen visit, the majority of the boys (*n* = 94, 81.7%) were at Tanner stage 1 for pubic hair development whereas 57 boys (49.6%) were at Tanner stage 1 for genital development. Most boys who were at Tanner stage 1 moved to more advanced Tanner stages at the late-teen visit- only 28 (26.4%) and 8 (7.5%) were still at Tanner stage 1 for pubic hair development and genital development after 3 years on average since the early-teen visits. Boys who were at Tanner stages 2, 3, and 4 at the early-teen visit all progressed to higher stages at the late-teen visit, with 14 (13.2%) and 18 (13.2%) boys reaching full development (Tanner stage = 5) for the two measurements. In terms of testicular volume distribution, the percentage of the boys in the prepubertal stage dropped from 14.8 to 0% from early-teen to late-teen visit.

### In utero and peripubertal metal exposure and peripubertal hormone concentrations

**Fig. 2:**
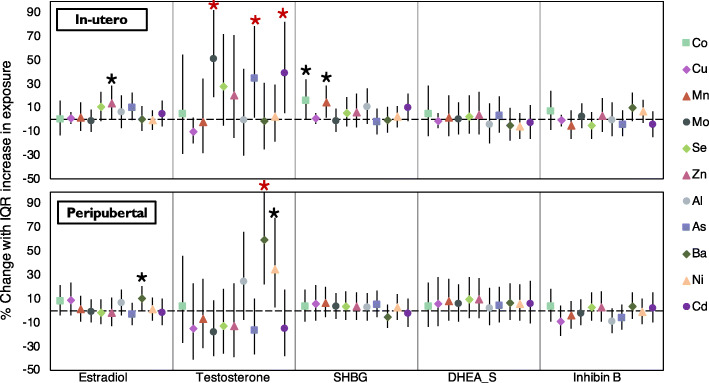
Peripubertal hormone concentrations associated with in utero and peripubertal metal concentration among ELEMENT boys^ab^. ^**a**^ Results are presented as the percent difference in peripubertal hormone concentrations associated with an interquartile range (IQR) increase in in utero and peripubertal metal. ^**b**^ Linear regression models were adjusted for child age, BMI z-score and specific gravity.  Significant associations with *p* value< 0.05 & *q* value< 0.15. * Significant associations with *p* value< 0.05 & *q* value> 0.15

Associations between in utero and peripubertal metal concentrations and reproductive hormones are presented in Fig. 2 and Supplementary Table S4. Positive associations were observed between some urinary essential metal concentrations and estradiol, testosterone, and SHBG. One IQR increase in in utero Zn concentration was associated with 13.7% higher serum estradiol (95% CI: 0.3, 28.8). In utero Co and Mn were positively associated with SHBG concentrations, with an IQR increase associated with 16% (95% CI:0.4, 34.2) and 14.2% (95%CI: 1.5, 28.5) higher serum SHBG after adjustment for child age, BMI z-score, and SG, respectively. As shown in Fig. 2, effect estimates for the association between both in utero essential and non-essential metal concentrations and testosterone were larger compared to other reproductive hormones, 51.3% for Mo, 35% for As, 38.9% for Cd. In models where reproductive hormones were regressed on concurrent peripubertal exposures, the strongest associations were observed also between metal concentrations and testosterone, particularly with non-essential metals Ni (%△/IQR: 35.1, 95% CI: 2.6, 77.8) and Ba (%△/IQR: 59.1, 95% CI: 22.5, 106.8). Peripubertal Ba concentration was also associated with higher estradiol (%△/IQR: 10.2, 95% CI: 0.5, 20.9). However, no significant associations were detected between peripubertal metal exposures and DHEA-S or inhibin B.

After correcting for multiple testing, the associations of in utero Mo, As, and Cd with testosterone, as well as the association of peripubertal Ba with testosterone had *q*-values < 0.15 (Fig. 2 and Supplementary Table S4), providing greater confidence in these associations.

### In utero and peripubertal metal exposure and sexual maturation

**Fig. 3:**
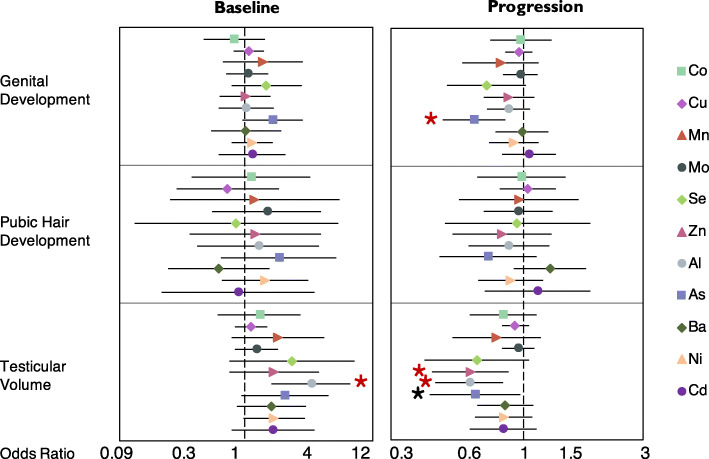
Odds Ratios (95% CI) for the Generalized Estimating Equations of in utero Metal Exposure and Tanner Stages/testicular volume^a^. ^**a**^ GEE models were adjusted for child age and BMI z-score (baseline and change).  Significant associations with *p* value< 0.05 & *q* value< 0.15. * Significant associations with *p* value< 0.05 & *q* value> 0.15

We have presented results from multiple ordinal regression models of in utero metal concentrations and Tanner stage and testicular volume in Fig. 3 and Supplementary Table S5. Tanner stages and testicular volume at the early-teen visit were not associated with in utero metal concentrations, with the exception of a non-essential metal, Al; an IQR increase in in utero Al concentrations was associated with 3.6 times greater odds (95% CI: 1.67, 7.76) of being at a higher category of testicular volume versus lower categories. In the same figure and table, associations between in utero metal concentrations and pubertal development over time in boys are also presented. During the follow-up, an IQR increase in in utero concentrations of non-essential metalloid As was associated with 36% (OR/IQR: 0.64, 95% CI: 0.48, 0.85) lower odds of genital development progression per year, adjusting for age, BMI, and Tanner stage at the early-teen visit (GEE longitudinal model). In utero concentrations of non-essential metal (loid) s Al (OR/IQR: 0.61, 95% CI: 0.45, 0.83) and As (OR/IQR: 0.64, 95% CI: 0.43, 0.97) were associated with lower odds of progressing to a higher testicular volume category (i.e. 39 and 36% lower odds/IQR). Essential metal Zn was also associated with 38% lower odds of testicular volume progression (OR/IQR: 0.62, 95% CI: 0.44, 0.88).

**Fig. 4:**
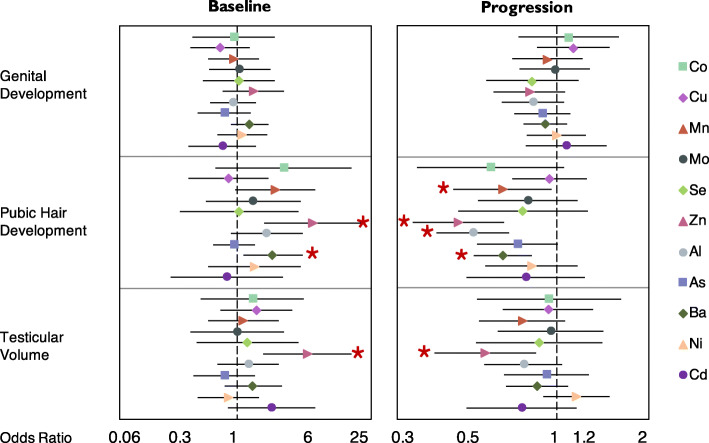
Odds Ratios (95% CI) for the Generalized Estimating Equations of peripubertal Metal Exposure and Tanner Stages/testicular volume^a^. ^**a**^ GEE models are adjusted for child age and BMI z-score (baseline and change).  Significant associations with *p* value< 0.05 & *q* value< 0.15. * Significant associations with *p* value< 0.05 & *q* value> 0.15

Similarly, Fig. 4 and Supplementary Table S5 show the cross-sectional and longitudinal associations between prepubertal metal concentrations and sexual maturation and progression. No significant associations were found between peripubertal urinary metal concentrations with Tanner stage for genital development or progression over follow-up, although several essential and non-essential metals were associated with pubic hair Tanner stages, testicular volume, and progressions. Peripubertal Zn concentration was associated with higher odds of being at a higher developmental stage for pubic hair (OR/IQR: 6.11, 95% CI: 1.89, 19.69) and testicular volume (OR/IQR: 5.39, 95% CI: 1.88, 15.49) at the early-teen visit, as well as slower progression of pubic hair development (OR/IQR: 0.47, 95% CI: 0.34, 0.67) and testicular volume (OR/IQR: 0.58, 95% CI: 0.40, 0.85) during the follow-up. Higher Mn was associated with 34% (OR/IQR: 0.66, 95% CI: 0.46, 0.96) lower pubic hair development progression only. Regarding non-essential metals, an IQR increase in peripubertal Ba concentration was associated with 2.3 times greater odds (95% CI: 1.15, 4.75) of being at higher Tanner stage for pubic hair development at age 8–14 years, but with 34% (OR/IQR: 0.66, 95% CI: 0.53, 0.83) lower odds of progressing to a higher pubic hair Tanner stage per year of follow-up. Higher peripubertal urinary Al was also associated with 47% (OR/IQR: 0.53, 95% CI: 0.40, 0.70) lower odds of pubic hair development progression per IQR increase.

All the associations described above had *q*-values < 0.15 (Fig. 3, Fig. 4, and Supplementary Table S5) after correcting for multiple testing, except for the association between in utero As and testicular volume progressions. Output graphics produced from GAM models showed that there was no significant non-linear relationship between essential and non-essential metal concentrations and hormones and sexual maturation outcomes, after adjusting for the same sets of covariates.

### Sensitivity analysis

Results from the hormone subset analysis when we restricted to boys who were prepubertal (94 out of 118) at the early-teen visit are similar to the main analysis results (Supplementary Table S6). For models regressing in utero metal concentrations, some effect estimates for DHEA_S, SHBG, and testosterone were no longer significant, potentially due to the small sample size. For models regressing peripubertal exposure, the notable effect estimates for testosterone were similar to those from the main analysis. The association between DHEA_S and Ni (%△/IQR =20.2, 95%CI = 1.7, 42.0) and inhibin B and Al (%△/IQR = -19.2, 95%CI = -31.7, − 4.4) became stronger and significant in the peripubertal subset analysis.

The magnitude of estimates from GEE models with and without BMI (Supplementary Table S7) were almost identical. Findings from models adjusting for SES were generally consistent with metal and hormone associations observed in our main analyses; in SES-adjusted models, the association between in utero Co concentrations and higher SHBG (%△/IQR = 14.2, 95%CI = -2.9, 23.3) was slightly attenuated and no longer significant, while the association between peripubertal Al and inhibin B (%△/IQR = -14.1, 95%CI = -24.6, − 2.2) was stronger and significant. In GEE models for Tanner stage or testicular volume status including SES finding remain consistent with the main models.

A high proportion of samples had metal concentrations above the LOD except for Cu, which was below the detection limit in 46% of samples. In a secondary analysis, we categorized urinary Cu concentrations into three groups. The low group consisted of values below the LOD, while the medium and high groups were made up of equalized bins among the detected values. We estimated the model parameters again and found that they were similar to the main parameter estimates.

## Discussion

Previous studies of metal exposure and male reproductive development have primarily been cross-sectional and focused on heavy metals, with the exception of “the Russian Children’s Study” and “Early Life Exposure in Mexico to Environmental Toxicants (ELEMENT) Study” that assessed the longitudinal impact of blood Pb [56, 63]. Thus, it is critical to evaluate the associations of essential and non-essential metals with reproductive hormones, sexual maturation, and progression among boys. Based on our longitudinal analysis of boys in a birth cohort in Mexico City who were examined at two peripubertal follow-up visits, we demonstrated a higher pubic hair development stage and testicular volume at the early-teen visit with higher non-essential metal concentrations, including in utero Al and peripubertal Ba and essential metal Zn concentration (peripubertal). We also found associations between in utero and peripubertal exposure to a number of both essential and non-essential metals and slowed progression of pubertal development across the follow-up period. In relation to hormone concentrations, higher non-essential metals, in utero As and Cd and peripubertal Ba concentrations as well as essential metal Mo measured in utero were associated with substantially higher testosterone. None of the associations between metals and hormones and measurements of sexual maturations appear to be non-linear.

To our knowledge, this is the first study to investigate the association between various in utero and peripubertal metals measured in urine and reproductive hormone concentrations in teenage boys. We reported a number of positive associations between specific metals measured in the urine and reproductive hormones, particularly testosterone. Some of our findings have not been reported elsewhere, such as relationships of essential minerals, e.g., Zn with estradiol or Co and Mn with SHBG. The positive association between relatively low Cd exposure and testosterone in this study is supported by epidemiological studies of non-occupational exposures among men [46, 48–50]. However, in vivo *and* in vitro observations linked Cd with altered adrenal gland activity and induced oxidative stress, which could result in toxic effects on the testis and subsequently decrease in serum testosterone [87–91].

Mo is an essential nutrient that is a cofactor for important enzymes involving in toxin removal [92, 93]. While no prior studies have investigated the associations between Mo and hormones in boys, contrary to our finding on in utero Mo and increased testosterone, decreased testosterone and sperm concentrations were observed in males with increasing levels of Mo [50, 60, 94]. Several animal studies have also reported the reproductive toxicity of Mo, including declines in sperm concentration, motility and normal morphology, testicular degeneration, and reduced fertility [95–98]. It is worth noting that the prenatal Mo concentrations were lower in our study population compared to the previous studies of Mo and reproductive outcomes and those reported in the National Health and Nutrition Examination Survey (NHANES) [99] (Supplementary Table S3). It is not clear whether Mo concentrations measured in utero will have different effects on reproductive hormones measured during different life stages, which warrants further investigation. Although previous studies have not evaluated associations between Ba and reproductive hormones in human, our finding of positive associations between peripubertal Ba concentrations and serum testosterone and estradiol is consistent with previous studies among male zebrafish, where exposure to barium chloride significantly increased estradiol concentrations and transcripts of genes involved in the HPG axis [100].

One of the main observations in this study is that Zn is associated with higher odds of pubic hair and testicular volume stage at the early-teen follow-up, but a slower progression of puberty from the early-teen to late-teen visit. Few cross-sectional studies on boys and girls also reported a significant positive relationship between serum or plasma zinc concentrations and stages of sexual maturation [101–104], as well as reproductive hormones (testosterone). Zn is an essential trace metal and is fundamental for the development of the male reproductive system [105, 106]. Studies on various animals described the role of Zn in advancing male puberty, through increased testicular activity, testosterone production, metabolism, and growth, primarily via activated the hypothalamus and the pituitary functions [107–112]. However, we did not observe a significant association between serum Zn and testosterone concentrations.

Pubic hair stages at the early-teen visit and progression were consistently associated with elevated concentrations of certain peripubertal metals, including As, Al, Mn, and Zn. The biological mechanisms that underlie the link between these essential and non-essential metals and pubertal development are uncertain but may be related to reproductive hormones. The main hormones responsible for pubic hair development in males are the androgens DHEA and testosterone [113, 114]. In this cohort, the strongest observed associations were between in utero and peripubertal metal concentrations and increased testosterone, while no significant associations were seen in relation to DHEA-S. It is possible that these metals impact the appearance of pubic hair through changes in testosterone.

In this study, we observed slower progression of sexual maturation for those boys at higher Tanner stages at the early-teen visit which was also reported in studies explored the association between pubertal onset and progression [115–117]. A compensatory mechanism similar to “catch-up growth” was proposed previously as a potential explanation for this observation [68, 70]. For those boys who had experienced a delayed pubertal development, their body systems may have responded by accelerating the tempo of pubertal progression (the change from lower stages of puberty to higher stages); while others who had experienced an advanced pubertal development may respond by slowing down the pubertal progression. This concept may explain the associations between metal exposure, earlier puberty onset at early-teen, and slower progression we found in this study. Future research is needed to establish this phenomenon and potential mechanisms.

Several of the associations with hormones and sexual maturation presented in this study are comparable to previous reports from human and animal research and supported by the current understanding of male pubertal development. However, there are inconsistent findings between the current study and previous studies and this may due to a number of reasons; 1) the exposure assessment approaches were different [sampling period, exposure matrices (i.e.*,* blood, urine, hair)]. 2) most of the previous studies exploring the relationship between metals and hormones were conducted on adult population. 3) most studies examined the cross-sectional relationships whereas we examined the longitudinal association between metals and sexual maturation and progression. It is also worth noting that the assessment of sexual maturation markers in this study was conducted by the same observers at both visits in this study to minimize measurement bias. Limitations of our study include a somewhat small sample size and few observations for certain Tanner stages, which may result in imprecise effect estimates. The age range of children at the early-teen and late-teen visits does overlap, however, our results are unlikely biased as the average follow up period for different age groups is 3.5 years. Some of the metals measured in this study have a relatively short half-life in urine, so urinary concentrations at the time measurement may not fully characterize exposure during each specific window of development. Lastly, hormone concentrations were only measured at one time point and are likely to be subject to non-differential misclassification due to diurnal variation. Further research is warranted to prospectively explore the underlying mechanisms by which metals may affect male sexual maturation and progression in larger study populations.

## Conclusions

The ELEMENT study, a prospective longitudinal birth cohort study in Mexico City, provided an opportunity to study the relationships of both in utero and peripubertal metal exposure on hormone concentrations and measures of sexual maturation and progression during the peripubertal period. Our results indicate that the in utero and peripubertal periods are vulnerable life stages, during which metal exposures may lead to disruption of male reproductive hormones and pubertal development. The findings also support that essential and non-essential metals have the potential to disrupt the onset and progression of puberty via interrupting the critical hormonal pathways.

## Supplementary Information

**Additional file 1.**

## Data Availability

All data generated and analyzed during this study are not publicly available due to the Institutional Review Board restrictions.
