# Supplementary material for: In utero and peripubertal metals exposure in relation to reproductive hormones and sexual maturation and progression among boys in Mexico City
Source: Environ Health. 2020 Nov 25;19:124. doi: 10.1186/s12940-020-00672-0 (PMC7688001; doi:10.1186/s12940-020-00672-0)

**Supplemental Information**

***In Utero* and Peripubertal Metal(loid)s Exposure in Relation to Reproductive Hormones and Sexual Maturation and Progression among boys in Mexico City**

Pahriya Ashrap, John D. Meeker, Brisa N. Sánchez, Niladri Basu, Marcela Tamayo-Ortiz, Maritsa Solano-González, Adriana Mercado-García, Martha M. Téllez-Rojo, Karen E. Peterson, Deborah J. Watkins^*^

**Table of Contents:**

| Table S1. Distribution of measures of sexual maturation at two visits among ELEMENT boys. | Page 2 |
| --- | --- |
| Table S2. Distribution of measures of sexual maturation at two visits for different age groups of ELEMENT boys. | Page 3-4 |
| Table S3. GM and median of uncorrected metal concentration among ELEMENT boys and NHANES (1997-2010) | Page 5-6 |
| Table S4. Percent difference in peripubertal hormone concentrations associated with an interquartile range (IQR) increase in *in utero* and peripubertal metal concentration among ELEMENT boys. | Page 7-8 |
| Table S5. Odds Ratios and 95% Confidence Intervals for the Ordinal Generalized Linear Regression of *in utero* and peripubertal Metal Exposure and Tanner Stage | Page 9-10 |
| Table S6. Percent difference in peripubertal hormone concentrations associated with an interquartile range (IQR) increase in *in utero* and peripubertal metal concentration, restricted to ELEMENT boys who were at pre-pubertal stage | Page 11-12 |
| Table S7. Odds Ratios and 95% Confidence Intervals for the Ordinal Generalized Linear Regression of *in utero* and peripubertal Metal Exposure and Tanner Stage, adjusted only for child age | Page 13-14 |
| Figure S1. Spaghetti plots for Tanner stages and testicular volume progression between early and late-teen visits | Page 15 |

Table S1. Distribution of age and measures of sexual maturation at two visits among ELEMENT boys.

| Secondary sex characteristics | Stages | Age (average, range) | Early-teen visit ^a^(N) | % | Age (average, range) | Late-teen visit ^b^ (N) | % |
| --- | --- | --- | --- | --- | --- | --- | --- |
| Pubic hair | 1 | 9.8 (8-14) | 94 | 81.7 | 11.9 (10-14) | 28 | 26.4 |
|  | 2 | 12.5 (11-14) | 17 | 14.8 | 12.9 (11-15) | 16 | 15.1 |
|  | 3 | 13.2 (12-14) | 3 | 2.6 | 13.7 (11-16) | 30 | 28.3 |
|  | 4 | 13.7 | 1 | 0.9 | 15.1 (13-18) | 18 | 17.0 |
|  | 5 | - | 0 | 0.0 | 15.9 (13-17) | 14 | 13.2 |
| Genitalia Development | 1 | 9.4 (8-11) | 57 | 49.6 | 11. 6 (10-12) | 8 | 7.5 |
|  | 2 | 10.7 (8-13) | 43 | 37.4 | 12.4 (11-13) | 17 | 16.0 |
|  | 3 | 12.6 (12-14) | 10 | 8.7 | 12.9 (11-16) | 26 | 24.5 |
|  | 4 | 13.3 (12-14) | 5 | 4.3 | 14.2 (12-16) | 37 | 34.9 |
|  | 5 | - | 0 | 0.0 | 15.8 (13-18) | 18 | 17.0 |
| Testicular Volume | <=3 ml | 9.6 (8-11) | 17 | 14.8 | - | 0 | 0.0 |
|  | 3~19 ml | 10.1 (8-14) | 85 | 73.9 | 12.6 (10-16) | 46 | 43.4 |
|  | >=20 ml | 12.8 (12-14) | 13 | 11.3 | 14.4 (11-18) | 60 | 56.6 |

^a^ total number of boys at early-teen visit (aged 8-14 years) =118 (with 3 boys missing info on sexual maturation measurements)
^b^ total number of boys at adolescence visit (aged 10-18 years) = 109 (with 3 boys missing info on sexual maturation measurements)

Table S2. Distribution of measures of sexual maturation at two visits for different age groups of ELEMENT boys.

| Secondary sex characteristics |  | Early-teen visit | | | | |  | Late-teen visit | | | | |
| --- | --- | --- | --- | --- | --- | --- | --- | --- | --- | --- | --- | --- |
|  | age | Stage 1  N (%) | Stage 2  N (%) | Stage 3 N (%) | Stage 4 N (%) | Stage 5 N (%) |  | Stage 1  N (%) | Stage 2 N (%) | Stage 3 N (%) | Stage 4 N (%) | Stage 5 N (%) |
|  | 8 | 8 (80) | 2 (20) | - | - | - |  | - | - | - | - | - |
| Pubic hair | 9 | 22 (71) | 9 (29) | - | - | - |  | - | - | - | - | - |
|  | 10 | 21 (72.4) | 8 (27.6) | - | - | - |  | 1 (100) | - | - | - | - |
|  | 11 | 5 (33.3) | 10 (66.7) | - | - | - |  | - | 3 (50) | 3 (50) | - | - |
|  | 12 | - | 11 (73.3) | 3 (20) | 1 (6.7) | - |  | 7 (31.8) | 5 (22.7) | 7 (31.8) | 3 (13.6) | - |
|  | 13 | - | 3 (27.3) | 6 (54.5) | 2 (18.2) | - |  | - | 8 (29.6) | 9 (33.3) | 8 (29.6) | 2 (7.4) |
|  | 14 | 1 (25) | - | 1 (25) | 2 (50) | - |  | - | 1 (5.6) | 4 (22.2) | 13 (72.2) | - |
|  | 15 | - | - | - | - | - |  | - | - | 1 (12.5) | 4 (50) | 3 (37.5) |
|  | 16 | - | - | - | - | - |  | - | - | 2 (10.5) | 8 (42.1) | 9 (47.4) |
|  | 17 | - | - | - | - | - |  | - | - | - | 1 (33.3) | 2 (66.7) |
|  | 18 | - | - | - | - | - |  | - | - | - | - | 2 (100) |
|  | age | Stage 1 N (%) | Stage 2 N (%) | Stage 3 N (%) | Stage 4 N (%) | Stage 5 N (%) |  | Stage 1 N (%) | Stage 2 N (%) | Stage 3 N (%) | Stage 4 N (%) | Stage 5 N (%) |
| Genitalia Development | 8 | 10 (100) | - | - | - | - |  | - | - | - | - | - |
|  | 9 | 31 (100) | - | - | - | - |  | - | - | - | - | - |
|  | 10 | 29 (100) | - | - | - | - |  | 1 (100) | - | - | - | - |
|  | 11 | 14 (93.3) | 1 (6.7) | - | - | - |  | 5 (83.3) | 1 (16.7) | - | - | - |
|  | 12 | 7 (46.7) | 7 (46.7) | 1 (6.7) | - | - |  | 15 (68.2) | 3 (13.6) | 4 (18.2) | - | - |
|  | 13 | 2 (18.2) | 8 (72.7) | 1 (9.1) | - | - |  | 5 (18.5) | 8 (29.6) | 10 (37) | 3 (11.1) | 1 (3.7) |
|  | 14 | 1 (25) | 1 (25) | 1 (25) | 1 (25) | - |  | 2 (11.1) | 3 (16.7) | 10 (55.6) | 3 (16.7) | - |
|  | 15 | - | - | - | - | - |  | - | 1 (12.5) | 1 (12.5) | 4 (50) | 2 (25) |
|  | 16 | - | - | - | - | - |  | - | - | 5 (26.3) | 6 (31.6) | 8 (42.1) |
|  | 17 | - | - | - | - | - |  | - | - | - | 1 (33.3) | 2 (66.7) |
|  | 18 | - | - | - | - | - |  | - | - | - | 1 (50) | 1 (50) |
|  | age | <=3 ml  N (%) | 3~19 ml  N (%) | >=20 ml  N (%) |  |  |  | <=3 ml  N (%) | 3~19 ml  N (%) | >=20 ml  N (%) |  |  |
| Testicular Volume | 8 | 3 (30) | 7 (70) | - |  |  |  | - | - | - |  |  |
|  | 9 | 7 (22.6) | 24 (77.4) | - |  |  |  | - | - | - |  |  |
|  | 10 | 5 (17.2) | 24 (82.8) | - |  |  |  | - | 1 (100) | - |  |  |
|  | 11 | 2 (13.3) | 13 (86.7) | - |  |  |  | - | 6 (100) | - |  |  |
|  | 12 | - | 11 (73.3) | 4 (26.7) |  |  |  | - | 16 (72.7) | 6 (27.3) |  |  |
|  | 13 | - | 5 (45.5) | 6 (54.5) |  |  |  | - | 14 (51.9) | 13 (48.1) |  |  |
|  | 14 | - | 1 (25) | 3 (75) |  |  |  | - | 4 (22.2) | 14 (77.8) |  |  |
|  | 15 | - | - | - |  |  |  | - | 2 (25) | 6 (75) |  |  |
|  | 16 | - | - | - |  |  |  | - | 2 (10.5) | 17 (89.5) |  |  |
|  | 17 | - | - | - |  |  |  | - | 1 (33.3) | 2 (66.7) |  |  |
|  | 18 | - | - | - |  |  |  | - | - | 2 (100) |  |  |

Table S3. GM and median urinary metal concentrations among ELEMENT boys and NHANES (1997-2010), uncorrected for specific gravity.

|  |  | **Prenatal** | | **Peripubertal** | |
| --- | --- | --- | --- | --- | --- |
| Metals | Cohort | GM | 50% | GM | 50% |
|  |  |  |  |  |  |
| **Co** | ELEMENT | 1.2 | 1.2 | 0.7 | 0.8 |
|  | NHANES | 0.4 | 0.4 | 0.5 | 0.5 |
| **Mn** | ELEMENT | 0.8 | 0.7 | 1.2 | 1.2 |
|  | NHANES**^a^** | 0.1 | 0.1 | 0.1 | 0.1 |
| **Mo** | ELEMENT | 19.5 | 25.7 | 46.6 | 50.2 |
|  | NHANES | 34.9~40.5 | 37.9~45.8 | 52.4~64.1 | 58.5~68.6 |
| **As** | ELEMENT | 14.0 | 13.2 | 14.3 | 14.4 |
|  | NHANES | 7.1~8.6 | 6.5~8.2 | 6.5~8.6 | 6.1~8.1 |
| **Ba** | ELEMENT | 4.0 | 4.2 | 2.5 | 2.4 |
|  | NHANES | 1.3~1.5 | 1.4~1.5 | 1.8~2.2 | 1.9~2.4 |
| **Cd** | ELEMENT | 0.2 | 0.2 | 0.1 | 0.1 |
|  | NHANES | 0.2 | 0.2 | 0.1 | 0.1 |

^a^ Mn was measured in NHANES cycle 2011-2012 and 2013- 2014.

^b^ Metal concentrations measured among female participants of NHANES was included in comparison for prenatal metal concentrations measured among participants in our study.

^c^ Metal concentrations measured among 12-19 years old children participants of NHANES were included in comparison for peripubertal metal concentrations measured among participants in our study.

Table S4. Percent difference in peripubertal hormone concentrations associated with an interquartile range (IQR) increase in *in utero* and peripubertal metal concentration among ELEMENT boys^a^.

|  | Estradiol | Testosterone | SHBG | DHEA-S | Inhibin B |
| --- | --- | --- | --- | --- | --- |
| *In utero* | %Δ/IQR^a^(95%CI) | %Δ/IQR^a^ (95%CI) | %Δ/IQR^a^ (95%CI) | %Δ/IQR^a^ (95%CI) | %Δ/IQR^a^ (95%CI) |
| *Essential metals* | |  |  |  |  |
| Co | 0.6 (-12.9, 16.1) | 5.1 (-28.7, 54.8) | **16.1 (0.4, 34.2)** | 5.1 (-14.2, 28.6) | 6.7 (-8.4, 24.3) |
| Cu | 1.4 (-3.1, 6.1) | -9.9 (-20.2, 1.8) | 0.9 (-3.7, 5.8) | -1.0 (-7.2, 5.5) | -0.7 (-5.4, 4.3) |
| Mn | 1.9 (-9.4, 14.6) | -1.8 (-28.4, 34.8) | **14.2 (1.5, 28.5)** | 1.9 (-13.6, 20.2) | -5.0 (-16.1, 7.6) |
| Mo | -1.1 (-10.0, 8.7) | **51.3 (19.1, 92.4)*** | -1.1 (-10.3, 9.0) | -0.1 (-12.5, 14.2) | 2.9 (-6.9, 13.8) |
| Se | 10.4 (-1.1, 23.2) | 27.8 (-5.0, 71.9) | 5.9 (-5.6, 18.7) | 2.9 (-12.0, 20.4) | -5.3 (-15.9, 6.6) |
| Zn | **13.7 (0.3, 28.8)** | 20.6 (-15.0, 71.3) | 6.6 (-6.7, 21.8) | 4.1 (-12.3, 23.5) | 3.1 (-10.3, 18.6) |
| *Non-essential metals* | |  |  |  |  |
| Al | 6.1 (-6.9, 20.8) | -0.4 (-30.5, 42.9) | 10.6 (-3.4, 26.7) | -4.4 (-19.7, 13.9) | -0.4 (-13.7, 14.9) |
| As | 10.3 (-0.7, 22.5) | **35.0 (1.8, 79.0)*** | -2.3 (-12.5, 9.1) | 3.2 (-11.2, 20.0) | -4.0 (-14.3, 7.6) |
| Ba | 0.3 (-9.6, 11.3) | -1.1 (-25.4, 31.1) | -0.8 (-10.9, 10.4) | -4.8 (-17.7, 10.2) | 10.0 (-1.4, 22.8) |
| Ni | -0.7 (-8.8, 8.0) | 2.7 (-18.3, 29.2) | 2.2 (-6.3, 11.5) | -6.0 (-16.5, 5.9) | 6.7 (-2.5, 16.6) |
| Cd | 4.8 (-5.6, 16.3) | **38.9 (5.6, 82.6)*** | 10 (-1.1, 22.3) | -2.8 (-16.1, 12.6) | -4.4 (-14.5, 6.8) |
|  |  |  |  |  |  |
| Peripubertal |  |  |  |  |  |
| *Essential metals* | |  |  |  |  |
| Co | 7.8 (-4.0, 21.2) | 3.6 (-26.5, 46.0) | 3.7 (-8.8, 18.0) | 3.4 (-13.3, 23.5) | 3.7 (-9.2, 18.4) |
| Cu | 9.0 (-3.8, 23.5) | -14.7 (-40.9, 23.1) | 5.6 (-8.1, 21.2) | 5.9 (-12.4, 28.0) | -9.2 (-21.2, 4.6) |
| Mn | 1.3 (-8.9, 12.7) | -6.8 (-31.6, 27.1) | 6.6 (-5.1, 19.7) | 8.0 (-7.9, 26.6) | -3.9 (-14.7, 8.4) |
| Mo | -0.9 (-10.0, 9.3) | -18.1 (-38.2, 8.6) | 3.5 (-7.0, 15.1) | 5.5 (-8.8, 22.2) | -2.1 (-12.3, 9.3) |
| Se | -1.4 (-11.2, 9.5) | -12.8 (-35.7, 18.3) | 3.6 (-7.7, 16.2) | 9.5 (-6.3, 28.1) | 2.9 (-8.6, 15.8) |
| Zn | 4.8 (-6.4, 17.3) | -2.2 (-28.3, 33.3) | -5.5 (-16.4, 6.8) | 6.3 (-9.3, 24.5) | 4.5 (-7.6, 18.2) |
| *Non-essential metals* | |  |  |  |  |
| Al | 6.7 (-3.5, 18.0) | 24.2 (-7.3, 66.5) | 2.9 (-7.9, 15.0) | 2.3 (-12.2, 19.2) | -9.0 (-18.8, 1.8) |
| As | -2.9 (-11.8, 6.8) | -16.7 (-36.9, 10.1) | 5.1 (-5.4, 16.8) | 4.2 (-9.9, 20.4) | -5.9 (-15.5, 4.8) |
| Ba | **10.2 (0.5, 20.9)** | **59.1 (22.5, 107)*** | -5.2 (-14.5, 5.2) | 6.6 (-7.5, 22.9) | 3.9 (-6.6, 15.6) |
| Ni | 1.2 (-8.1, 11.4) | **35.1 (2.6, 77.8)** | 2.5 (-7.7, 14) | 5.8 (-8.4, 22.2) | -1.0 (-11.2, 10.3) |
| Cd | -1.6 (-11.9, 10.0) | -14.7 (-38.3, 17.9) | -2.3 (-13.5, 10.4) | 5.8 (-10.5, 25.1) | 2.0 (-10.0, 15.7) |

**^a^** Linear regression models were adjusted for child age, BMI z-score and specific gravity

**q* value (false discovery rate) <0.15

Table S5. Odds Ratios and 95% Confidence Intervals for the Ordinal Generalized Linear Regression of *in utero* and peripubertal Metal Exposure and Tanner Stage^a^

|  |  | ***In utero* exposure** | | | **Peripubertal exposure** | | |
| --- | --- | --- | --- | --- | --- | --- | --- |
|  | **Effect^b^** | **Genital**  **Development** | **Pubic Hair Development** | **Testicular Volume** | **Genital**  **Development** | **Pubic Hair Development** | **Testicular**  **Volume** |
|  |  | **OR/IQR^c^ (95%CI)** | **OR/IQR^c^ (95%CI)** | **OR/IQR^c^ (95%CI)** | **OR/IQR^c^ (95%CI)** | **OR/IQR^c^ (95%CI)** | **OR/IQR^c^ (95%CI)** |
| *Essential metals* | |  |  |  |  |  |  |
| **Co** | Cross-sectional | 0.82 (0.46, 1.47) | 1.14 (0.36, 3.58) | 1.33 (0.6, 2.96) | 0.92 (0.34, 2.48) | 3.05 (0.6, 15.52) | 1.44 (0.42, 4.94) |
|  | Tempo | 0.98 (0.74, 1.30) | 0.98 (0.66, 1.47) | 0.83 (0.61, 1.13) | 1.09 (0.75, 1.59) | 0.60 (0.35, 1.05) | 0.94 (0.55, 1.62) |
| **Cu** | Cross-sectional | 1.09 (0.81, 1.46) | 0.72 (0.27, 1.94) | 1.14 (0.84, 1.55) | 0.66 (0.33, 1.35) | 0.81 (0.31, 2.09) | 1.59 (0.67, 3.76) |
|  | Tempo | 0.96 (0.85, 1.09) | 1.04 (0.81, 1.35) | 0.93 (0.82, 1.05) | 1.13 (0.86, 1.49) | 0.94 (0.71, 1.25) | 0.93 (0.66, 1.32) |
| **Mn** | Cross-sectional | 1.43 (0.66, 3.10) | 1.21 (0.24, 6.21) | 1.92 (0.78, 4.70) | 0.92 (0.49, 1.71) | 2.49 (0.95, 6.52) | 1.15 (0.49, 2.68) |
|  | Tempo | 0.81 (0.57, 1.15) | 0.96 (0.56, 1.66) | 0.78 (0.53, 1.17) | 0.93 (0.71, 1.21) | **0.66 (0.46, 0.96)*** | 0.77 (0.55, 1.07) |
| **Mo** | Cross-sectional | 1.06 (0.71, 1.60) | 1.54 (0.54, 4.43) | 1.25 (0.83, 1.89) | 1.06 (0.51, 2.21) | 1.46 (0.47, 4.54) | 1.00 (0.33, 3.04) |
|  | Tempo | 0.97 (0.83, 1.14) | 0.95 (0.69, 1.30) | 0.95 (0.82, 1.10) | 0.98 (0.75, 1.28) | 0.80 (0.55, 1.16) | 0.95 (0.64, 1.41) |
| **Se** | Cross-sectional | 1.53 (0.77, 3.01) | 0.86 (0.12, 6.13) | 2.50 (0.75, 8.32) | 1.04 (0.44, 2.46) | 1.04 (0.25, 4.30) | 1.28 (0.38, 4.31) |
|  | Tempo | 0.71 (0.50, 1.02) | 0.95 (0.49, 1.84) | 0.65 (0.41, 1.05) | 0.83 (0.58, 1.17) | 0.77 (0.47, 1.26) | 0.87 (0.54, 1.41) |
| **Zn** | Cross-sectional | 1.02 (0.62, 1.66) | 1.23 (0.35, 4.35) | 1.76 (0.74, 4.18) | 1.47 (0.70, 3.09) | **6.11 (1.89, 19.69)*** | **5.39 (1.88, 15.49)*** |
|  | Tempo | 0.88 (0.70, 1.10) | 0.82 (0.52, 1.29) | **0.62 (0.44, 0.88)*** | 0.81 (0.62, 1.06) | **0.47 (0.34, 0.67)*** | **0.58 (0.40, 0.85)*** |
|  | |  |  |  |  |  |  |
| *Non-essential metals* | |  |  |  |  |  |  |
| **Al** | Cross-sectional | 1.03 (0.60, 1.75) | 1.31 (0.40, 4.24) | **3.6 (1.67, 7.76)*** | 0.91 (0.52, 1.58) | 2.03 (0.85, 4.84) | 1.31 (0.63, 2.74) |
|  | Tempo | 0.87 (0.71, 1.06) | 0.88 (0.60, 1.27) | **0.61 (0.45, 0.83)*** | 0.83 (0.66, 1.05) | **0.53 (0.40, 0.70)*** | 0.77 (0.58, 1.04) |
| **As** | Cross-sectional | 1.71 (0.95, 3.07) | 1.93 (0.63, 5.91) | 2.18 (0.94, 5.08) | 0.74 (0.39, 1.39) | 0.93 (0.57, 1.52) | 0.74 (0.35, 1.53) |
|  | Tempo | **0.64 (0.48, 0.85)*** | 0.73 (0.47, 1.13) | **0.64 (0.43, 0.97)** | 0.90 (0.73, 1.11) | 0.74 (0.54, 1.00) | 0.92 (0.67, 1.28) |
| **Ba** | Cross-sectional | 1.03 (0.53, 2.03) | 0.61 (0.23, 1.62) | 1.69 (0.87, 3.27) | 1.34 (0.86, 2.10) | **2.33 (1.15, 4.75)*** | 1.47 (0.75, 2.88) |
|  | Tempo | 0.99 (0.78, 1.26) | 1.28 (0.92, 1.78) | 0.85 (0.65, 1.10) | 0.92 (0.78, 1.08) | **0.66 (0.53, 0.83)*** | 0.86 (0.68, 1.09) |
| **Ni** | Cross-sectional | 1.17 (0.79, 1.73) | 1.49 (0.65, 3.45) | 1.77 (0.98, 3.20) | 1.14 (0.62, 2.08) | 1.51 (0.50, 4.59) | 0.81 (0.39, 1.69) |
|  | Tempo | 0.92 (0.73, 1.15) | 0.89 (0.66, 1.20) | 0.84 (0.64, 1.09) | 0.99 (0.8, 1.24) | 0.82 (0.58, 1.17) | 1.16 (0.90, 1.49) |
| **Cd** | Cross-sectional | 1.16 (0.61, 2.20) | 0.88 (0.20, 3.86) | 1.74 (0.78, 3.88) | 0.70 (0.31, 1.57) | 0.78 (0.20, 2.98) | 2.28 (0.81, 6.44) |
|  | Tempo | 1.05 (0.82, 1.34) | 1.14 (0.71, 1.84) | 0.83 (0.61, 1.13) | 1.07 (0.79, 1.45) | 0.79 (0.51, 1.23) | 0.76 (0.50, 1.15) |

**^a^** GEE models were adjusted for child age and BMI z-score (baseline and change)

^b^ The effects are cross-sectional effect of metal on Tanner stage/testicular volume and the effect of metal on the progression of Tanner stage/testicular volume

^c^ Odds ratio associated with an IQR increase in metal concentration.

**q* value (false discovery rate) <0.15

Table S6. Percent difference in peripubertal hormone concentrations associated with an interquartile range (IQR) increase in *in utero* and peripubertal metal concentration, restricted to ELEMENT **boys at pubic hair stage 1^a^**.

|  | Estradiol | Testosterone | SHBG | DHEA-S | Inhibin B |
| --- | --- | --- | --- | --- | --- |
| *In utero* | %Δ/IQR^a^(95%CI) | %Δ/IQR^a^ (95%CI) | %Δ/IQR^a^ (95%CI) | %Δ/IQR^a^ (95%CI) | %Δ/IQR^a^ (95%CI) |
| *Essential metals* | |  |  |  |  |
| Co | 5.9 (-14.1, 30.6) | -1.5 (-43.2, 70.9) | 18.5 (-4.1, 46.6) | -4.3 (-29.1, 29.2) | 6.2 (-15.5, 33.4) |
| Cu | 0.8 (-11.4, 14.7) | **-33.7 (-51.7, -9.0)*** | 5.2 (-7.9, 20.1) | -2.4 (-18.8, 17.4) | -2.6 (-15.4, 12.1) |
| Mn | 3.2 (-10.8, 19.4) | **-35.3 (-55, -7.1)*** | 13.9 (-1.7, 31.9) | -5.8 (-23.5, 16) | -12.1 (-24.8, 2.6) |
| Mo | 0.8 (-18.6, 24.8) | **119.4 (31.5, 266)*** | -10.5 (-28.1, 11.4) | 3.0 (-24.1, 39.7) | 0.5 (-20.4, 26.8) |
| Se | 3.7 (-10.6, 20.2) | 0.5 (-31.9, 48.1) | 5.0 (-9.9, 22.3) | 11.3 (-9.7, 37.2) | -8.9 (-22.3, 6.8) |
| Zn | 5.5 (-13.1, 28.0) | -13.2 (-45.4, 37.9) | 3.0 (-15.5, 25.5) | 2.3 (-20.7, 31.9) | 4.8 (-14.3, 28) |
| *Non-essential metals* | |  |  |  |  |
| Al | 2.2 (-15.2, 23.2) | -27.1 (-52.8, 12.8) | 10.2 (-8.6, 32.9) | -8.7 (-28.3, 16.3) | -2.9 (-19.9, 17.8) |
| As | **15.3 (1.0, 31.6)** | 5.4 (-26.7, 51.6) | 1.8 (-11.8, 17.5) | 3.3 (-15.3, 25.9) | -6.4 (-19.4, 8.7) |
| Ba | 3.2 (-10.7, 19.2) | 3.9 (-28.9, 51.7) | 3.7 (-10.7, 20.4) | -5.8 (-23.3, 15.7) | 11.5 (-4.5, 30.1) |
| Ni | -0.3 (-13.3, 14.6) | -15.6 (-41.3, 21.2) | 0.5 (-13.1, 16.1) | -2.3 (-19.9, 19.3) | 2.6 (-11.9, 19.4) |
| Cd | 7.0 (-4.9, 20.4) | 1.6 (-25.8, 38.9) | 1.4 (-10.4, 14.8) | 2.5 (-13.6, 21.6) | -4.3 (-16, 8.9) |
|  |  |  |  |  |  |
| Peripubertal |  |  |  |  |  |
| *Essential metals* | |  |  |  |  |
| Co | 17.7 (-4.2, 44.7) | 16.1 (-32.9, 101) | 0.9 (-19.1, 25.9) | 5.4 (-21.8, 42.2) | -2.4 (-23.4, 24.4) |
| Cu | 15.4 (-6.4, 42.3) | -26.5 (-57.6, 27.3) | 12.3 (-10, 40.1) | -9.5 (-33.1, 22.4) | -12.1 (-31.1, 12) |
| Mn | 2.5 (-11.2, 18.4) | -1.1 (-31.9, 43.8) | 10.3 (-4.8, 27.9) | 8.6 (-11.3, 32.9) | -9.8 (-23.3, 6.1) |
| Mo | 7.1 (-8.7, 25.5) | -7.4 (-38.9, 40.5) | 0.4 (-15.1, 18.7) | 5.2 (-16.1, 32) | -7.6 (-23, 11) |
| Se | 10.8 (-6.2, 30.9) | 4.0 (-33, 61.5) | 3.4 (-13.3, 23.4) | -1.0 (-22.1, 25.8) | 3.6 (-14.6, 25.8) |
| Zn | 11.7 (-6, 32.8) | -14.8 (-45.1, 32.2) | -8.4 (-23.6, 9.8) | -2.5 (-23.7, 24.6) | 8.1 (-10.2, 30.2) |
| *Non-essential metals* | |  |  |  |  |
| Al | 8.3 (-7.1, 26.2) | 32 (-11.2, 96.2) | 14.8 (-1.9, 34.5) | -6.4 (-24.8, 16.6) | **-19.2 (-31.7, -4.4)** |
| As | -3.2 (-18.2, 14.7) | -11.7 (-43.1, 37) | -2.8 (-18.6, 16) | 16.4 (-8.1, 47.4) | -5.4 (-22.1, 14.8) |
| Ba | 5.3 (-9.3, 22.2) | **50.8 (3.9, 119)** | -12.5 (-24.9, 1.9) | 19.4 (-2.9, 46.8) | -5.4 (-20.3, 12.3) |
| Ni | 4.5 (-7.6, 18.1) | **40.1 (3.1, 90.2)** | -9.2 (-20, 2.9) | **20.2 (1.7, 42)** | -6.7 (-18.9, 7.3) |
| Cd | -3.6 (-22.4, 19.8) | -11.5 (-49.7, 55.9) | -4.1 (-23.6, 20.4) | -0.3 (-26.8, 35.7) | 8.7 (-15.3, 39.4) |

**^a^** Linear regression models were adjusted for child age, BMI z-score and specific gravity

**q* value (false discovery rate) <0.15

Table S7. Odds ratios and 95% confidence intervals for the ordinal generalized linear regression of *in utero* and peripubertal metal exposure and Tanner stage, adjusted only for child age^a^

|  |  | ***In utero* exposure** | | | **Peripubertal exposure** | | |
| --- | --- | --- | --- | --- | --- | --- | --- |
|  | **Effect^b^** | **Genital Development** | **Pubic Hair Development** | **Testicular Volume** | **Genital Development** | **Pubic Hair Development** | **Testicular Volume** |
|  |  | **OR/IQR^c^ (95%CI)** | **OR/IQR^c^ (95%CI)** | **OR/IQR^c^ (95%CI)** | **OR/IQR^c^ (95%CI)** | **OR/IQR^c^ (95%CI)** | **OR/IQR^c^ (95%CI)** |
| *Essential metals* | |  |  |  |  |  |  |
| **Co** | Cross-sectional | 0.78 (0.45, 1.35) | 1.15 (0.38, 3.53) | 1.32 (0.59, 2.96) | 0.87 (0.31, 2.41) | 3.03 (0.61, 15.04) | 1.49 (0.43, 5.13) |
|  | Tempo | 0.97 (0.73, 1.28) | 0.95 (0.64, 1.42) | 0.85 (0.63, 1.14) | 1.09 (0.74, 1.59) | 0.60 (0.35, 1.04) | 0.95 (0.55, 1.63) |
| **Cu** | Cross-sectional | 1.04 (0.78, 1.38) | 0.70 (0.26, 1.88) | 1.14 (0.84, 1.54) | 0.67 (0.32, 1.40) | 0.81 (0.32, 2.09) | 1.56 (0.67, 3.68) |
|  | Tempo | 0.97 (0.86, 1.11) | 1.05 (0.81, 1.37) | 0.93 (0.82, 1.05) | 1.15 (0.87, 1.51) | 0.95 (0.71, 1.26) | 0.91 (0.66, 1.25) |
| **Mn** | Cross-sectional | 1.41 (0.65, 3.05) | 1.23 (0.24, 6.37) | 1.91 (0.79, 4.66) | 0.96 (0.52, 1.80) | 2.45 (0.94, 6.36) | 1.13 (0.48, 2.67) |
|  | Tempo | 0.83 (0.59, 1.17) | 0.95 (0.55, 1.65) | 0.79 (0.53, 1.17) | 0.95 (0.73, 1.23) | **0.67 (0.46, 0.96)*** | 0.75 (0.54, 1.03) |
| **Mo** | Cross-sectional | 1.12 (0.76, 1.66) | 1.65 (0.60, 4.57) | 1.25 (0.83, 1.87) | 1.14 (0.55, 2.34) | 1.40 (0.47, 4.17) | 0.99 (0.33, 2.97) |
|  | Tempo | 0.96 (0.82, 1.12) | 0.93 (0.68, 1.26) | 0.96 (0.83, 1.10) | 0.98 (0.75, 1.28) | 0.80 (0.55, 1.16) | 0.94 (0.64, 1.37) |
| **Se** | Cross-sectional | 1.53 (0.78, 3.00) | 0.91 (0.13, 6.42) | 2.49 (0.76, 8.22) | 1.02 (0.42, 2.46) | 1.02 (0.25, 4.14) | 1.28 (0.38, 4.30) |
|  | Tempo | 0.73 (0.51, 1.04) | 0.92 (0.48, 1.78) | 0.66 (0.41, 1.06) | 0.82 (0.58, 1.16) | 0.77 (0.48, 1.25) | 0.89 (0.56, 1.41) |
| **Zn** | Cross-sectional | 1.07 (0.65, 1.76) | 1.29 (0.37, 4.53) | 1.74 (0.74, 4.07) | 1.46 (0.69, 3.08) | **6.22 (1.92, 20.18)*** | **5.34 (1.84, 15.44)*** |
|  | Tempo | 0.87 (0.69, 1.09) | 0.80 (0.52, 1.25) | **0.63 (0.44, 0.90)*** | 0.82 (0.62, 1.07) | **0.47 (0.34, 0.67)*** | **0.58 (0.40, 0.86)*** |
|  |  |  |  |  |  |  |  |
| *Non-essential metals* | |  |  |  |  |  |  |
| **Al** | Cross-sectional | 0.95 (0.56, 1.63) | 1.29 (0.39, 4.22) | **3.62 (1.67, 7.83)*** | 0.90 (0.52, 1.56) | 2.01 (0.84, 4.83) | 1.31 (0.63, 2.71) |
|  | Tempo | 0.90 (0.74, 1.09) | 0.87 (0.60, 1.27) | **0.61 (0.45, 0.84)*** | 0.84 (0.67, 1.06) | **0.53 (0.41, 0.70)*** | 0.77 (0.57, 1.03) |
| **As** | Cross-sectional | 1.74 (0.99, 3.06) | 1.95 (0.64, 5.91) | 2.18 (0.94, 5.07) | 0.80 (0.42, 1.53) | 0.92 (0.56, 1.52) | 0.73 (0.35, 1.52) |
|  | Tempo | **0.64 (0.49, 0.84)*** | 0.70 (0.46, 1.09) | **0.65 (0.43, 0.99)** | 0.88 (0.71, 1.09) | 0.74 (0.55, 1.01) | 0.94 (0.68, 1.29) |
| **Ba** | Cross-sectional | 1.05 (0.55, 2.01) | 0.65 (0.25, 1.73) | 1.67 (0.88, 3.20) | 1.26 (0.79, 2.00) | **2.36 (1.16, 4.79)*** | 1.48 (0.75, 2.93) |
|  | Tempo | 1.00 (0.79, 1.27) | 1.25 (0.90, 1.74) | 0.85 (0.66, 1.10) | 0.92 (0.78, 1.08) | **0.66 (0.53, 0.82)*** | 0.86 (0.68, 1.09) |
| **Ni** | Cross-sectional | 1.13 (0.75, 1.70) | 1.55 (0.67, 3.58) | 1.75 (0.96, 3.20) | 1.13 (0.61, 2.09) | 1.49 (0.49, 4.51) | 0.80 (0.38, 1.69) |
|  | Tempo | 0.91 (0.73, 1.14) | 0.87 (0.65, 1.16) | 0.84 (0.65, 1.09) | 1.01 (0.81, 1.26) | 0.83 (0.59, 1.18) | 1.12 (0.87, 1.44) |
| **Cd** | Cross-sectional | 1.17 (0.62, 2.22) | 0.93 (0.21, 4.10) | 1.72 (0.79, 3.78) | 0.75 (0.34, 1.67) | 0.78 (0.21, 2.93) | 2.24 (0.80, 6.31) |
|  | Tempo | 1.04 (0.82, 1.31) | 1.10 (0.68, 1.79) | 0.84 (0.62, 1.14) | 1.05 (0.78, 1.42) | 0.79 (0.51, 1.23) | 0.77 (0.51, 1.15) |

**^a^** GEE models were adjusted for only child age

^b^ The effects are cross-sectional effect of metal on Tanner stage/testicular volume and the effect of metal on the progression of Tanner stage/testicular volume

^b^ Odds ratio associated with an IQR increase in metal concentration.

**q* value (false discovery rate) <0.15

Figure S1. Spaghetti plots for Tanner stages and testicular volume progression between early and late-teen visits


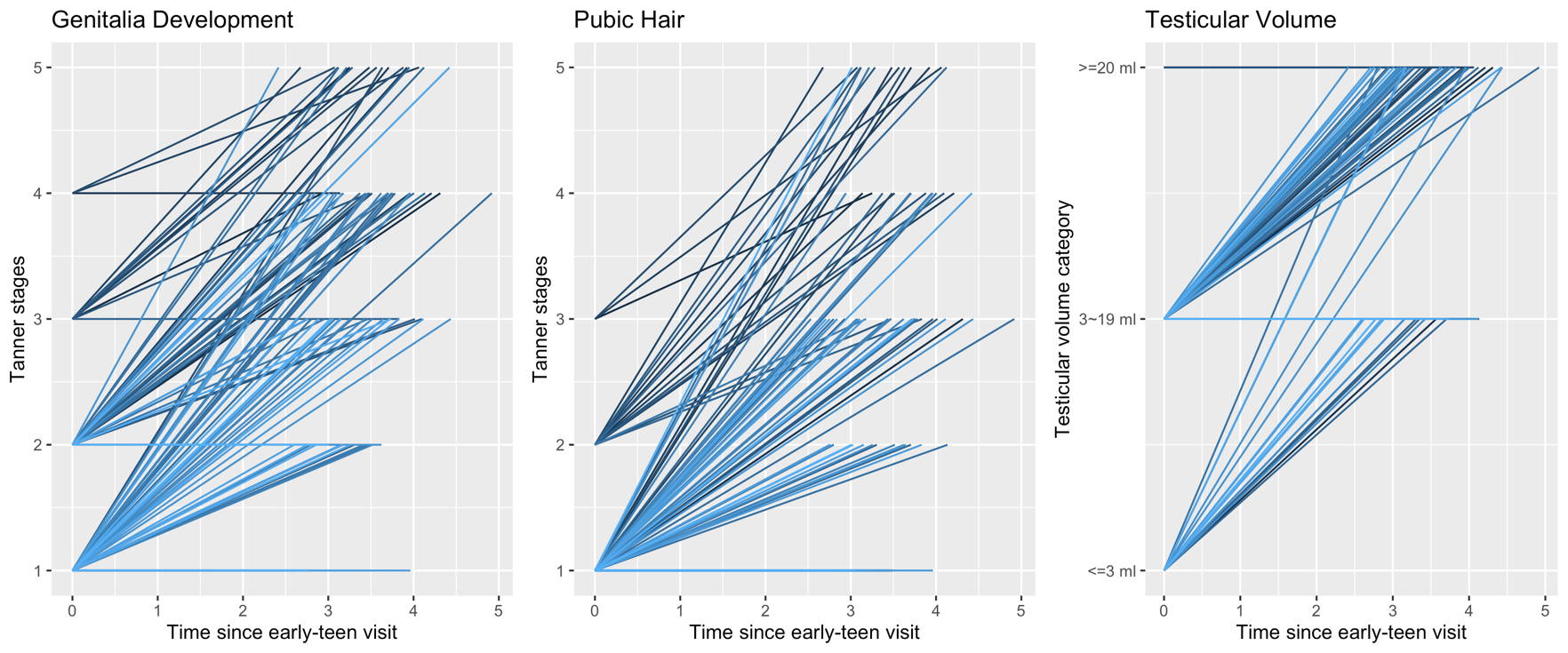

Supplement: Supplementary file 1 — Additional file 1. [file 12940_2020_672_MOESM1_ESM.docx]
